# Wastewater-based surveillance models for COVID-19: A focused review on spatio-temporal models

**DOI:** 10.1016/j.heliyon.2023.e21734

**Published:** 2023-11-08

**Authors:** Fatemeh Torabi, Guangquan Li, Callum Mole, George Nicholson, Barry Rowlingson, Camila Rangel Smith, Radka Jersakova, Peter J. Diggle, Marta Blangiardo

**Affiliations:** a Turing-RSS Health Data Lab, London, UK; b Population Data Science HDRUK-Wales, Medical School, Swansea University, Wales, UK; c Applied Statistics Research Group, Department of Mathematics, Physics and Electrical Engineering, Northumbria University, Newcastle upon Tyne NE1 8ST, UK; d The Alan Turing Institute, London, UK; e University of Oxford, Oxford, UK; f CHICAS, Lancaster Medical School, Lancaster University, England, UK; g MRC Centre for Environment and Health, Department of Epidemiology and Biostatistics, Imperial College, London, UK

**Keywords:** Wastewater-based surveillance, Wastewater-based epidemiology, COVID-19, Spatio-temporal statistical modelling

## Abstract

The evident shedding of the SARS-CoV-2 RNA particles from infected individuals into the wastewater opened up a tantalizing array of possibilities for prediction of COVID-19 prevalence prior to symptomatic case identification through community testing. Many countries have therefore explored the use of wastewater metrics as a surveillance tool, replacing traditional direct measurement of prevalence with cost-effective approaches based on SARS-CoV-2 RNA concentrations in wastewater samples. Two important aspects in building prediction models are: time over which the prediction occurs and space for which the predicted case numbers is shown. In this review, our main focus was on finding mathematical models which take into the account both the time-varying and spatial nature of wastewater-based metrics into account. We used six main characteristics as our assessment criteria: i) modelling approach; ii) temporal coverage; iii) spatial coverage; iv) sample size; v) wastewater sampling method; and vi) covariates included in the modelling. The majority of studies in the early phases of the pandemic recognized the temporal association of SARS-CoV-2 RNA concentration level in wastewater with the number of COVID-19 cases, ignoring their spatial context. We examined 15 studies up to April 2023, focusing on models considering both temporal and spatial aspects of wastewater metrics. Most early studies correlated temporal SARS-CoV-2 RNA levels with COVID-19 cases but overlooked spatial factors. Linear regression and SEIR models were commonly used (n = 10, 66.6 % of studies), along with machine learning (n = 1, 6.6 %) and Bayesian approaches (n = 1, 6.6 %) in some cases. Three studies employed spatio-temporal modelling approach (n = 3, 20.0 %). We conclude that the development, validation and calibration of further spatio-temporally explicit models should be done in parallel with the advancement of wastewater metrics before the potential of wastewater as a surveillance tool can be fully realised.

## Highlights

• Many countries explored relationship of SARS-CoV-2 RNA concentrations in wastewater samples and COVID-19 cases.
• Wastewater metrics can potentially be used as an early warning tool for the pandemic.
• Most of the published work does not take account of the spatial context.
• There is a high spatial heterogeneity in COVID-19 prevalence.
• Development of models capturing spatio-temporal relationship of WW metrics are needed.

## Graphical abstract

demonstrating the role and stage of wastewater-based modelling in the whole cycle of epidemic surveillance.

**Figure:**
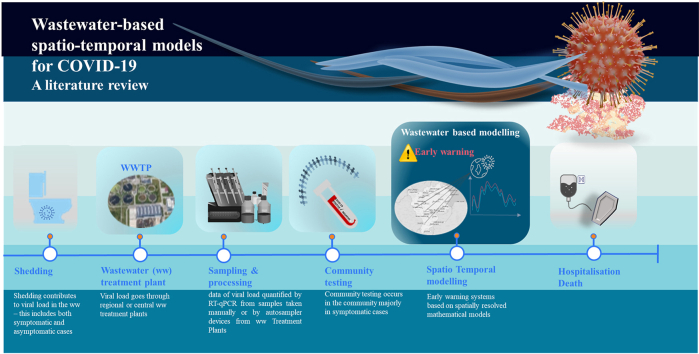

## 1 Introduction

### 1.1 Traditional surveillance tools

Epidemic surveillance traditionally uses a combination of case-reporting and sample surveys to monitor the evolving pattern of disease over time and space. Traditional surveillance often focuses on certain geographical areas resulting in limited coverage and lack of ability to capture mobility; additionally, it can be very expensive and resource-intensive and often can lack real-time data as operating on the basis of data gathered through set protocols with a time lag. In the case of COVID-19, in addition to the above limitations, the traditional surveillance methods are also limited to symptomatic cases who get tested and receive a positive COVID-19 test, which is a biased estimate of the infected population at each point in time. For a disease with low mortality rate, case-reporting tends to be incomplete, but this can be addressed by analysing case-reporting data jointly with data from randomised, and therefore unbiased, prevalence surveys [1,2]. During the COVID-19 pandemic, national and international health agencies have developed surveillance tools, a very well-known exemplar was the World Health Organisation dashboard [3], for monitoring the pandemic status. Other examples include (i) the COVID Map developed by Johns Hopkins coronavirus resource centre providing global weekly statistics since March 2020 [4]; (ii) the COVID Data Tracker tool developed by the US Centers for Disease Control and Prevention (CDC) providing daily statistics on COVID-19 in the USA [5]; (iii) the COVID-19 dashboard providing daily statistics on the status of the pandemic across the UK [6]; (iv) a non-government initiative by Zoe Global Limited and King's College London, for daily tracking of self-reported COVID-19 symptoms via a mobile phone app [7,8].

The global capacity for real-time disease surveillance varies among countries, as has been partially demonstrated by analysis of self-reported data on detection, control and prevention of outbreaks across 182 countries [9]. Detection and surveillance in most Low and Middle Income Countries (LMIC) has relied on existing testing capacities [10,11]. In the UK, the two largest national randomised studies of COVID-19 prevalence were run by Imperial College London and the Office for National Statistics (ONS). The Imperial College REal-time Assessment of Community Transmission (REACT) study used a repeated cross-sectional study-design, with 19 rounds of data-collection, at approximately monthly intervals between May 2020 and March 2022 [12], recruiting approximately 100,000 individuals in each round by stratified random sampling from 315 local authorities in the National Health Service list for England [13]. The ONS COVID-19 Infection Survey (CIS) used a longitudinal sampling of households, constituting a total of approximately 150,000 individuals, until July 14, 2022 [14].

The great advantage of a randomised study-design is the guarantee of unbiasedness, albeit with the proviso that non-compliance must be assumed to be ignorable, by which we mean that the fact of a person's non-compliance is independent of their COVID-19 infection status conditional on all of their measured characteristics [15]. The main disadvantage is the relatively high cost, when compared with routinely collected or self-reported data-streams. This raises the question of whether the cost-effectiveness of a surveillance system might be maximised by a joint analysis of data from a relatively small randomised prevalence study and one or more large-scale, low-cost data-sources whose outputs are predictive of disease prevalence. The shortcomings of traditional surveillance methods such as lag in reporting and cost efficiency can be tackled by combining existing data with readily accessible wastewater metrics. The choice of sampling locations in wastewater-based surveillance can easily be adapted over time in response to changing epidemic dynamics; additionally, all infected (symptomatic and asymptomatic) will be contributing to the viral load concentration in wastewater. Also, while prediction at fine spatial resolution can only be achieved in high-income countries, most countries across the globe have capacities to implement these measures at regional level [10,11].

### 1.2 Wastewater for epidemic surveillance

Wastewater has been used in epidemic surveillance and to create tools for monitoring infectious disease [16] and drug-abuse [17,18]. Other potential public health applications of wastewater-based epidemiology (WBE) include food biomarkers, antibiotic resistance and many more [19]. Early in the pandemic, reports confirmed the detectability of RNA particles of severe acute respiratory syndrome coronavirus 2 (SARS-CoV-2), the causal agent of COVID-19, in intestine and stool samples of infected individuals [20]. In September 2020, an analysis of wastewater sludge, the more concentrated solid part of a wastewater sample, showed that early infection signals could be obtained by monitoring the viral load in wastewater [[21], [22], [23], [24]]. Association between viral load in wastewater samples and COVID-19 prevalence in the community has been documented [25]. In addition, WBE has the potential to provide an early warning system that can be used for implementation of timely containment measures for early detection of changes [[26], [27], [28]]. Reports on wastewater as an early detection tool show a potential to achieve an early signal with 1–14 days lead over traditional disease metrics, dependent on the phase of the pandemic and on the accessibility of clinical testing [29]. For instance, early detection of Alpha and Omicron variants of concern have been achieved through wastewater metrics [[30], [31], [32]]. There are also reports on early signals from wastewater during the first phase of the pandemic [33].

### 1.3 Wastewater modelling

Globally, there are more than 3000 active wastewater testing sites located across 58 countries [34]. The majority (85 %) of the currently identified sites are located in high-to middle-income countries. Nevertheless, a variety of modelling approaches have been developed and applied in many countries [[35], [36], [37], [38], [39], [40], [41], [42], [43]](Table S3).

Despite all the progress in this area, using RNA viral load, the main source of the SARS-CoV-2 signal in wastewater, as a proxy for disease prevalence raises some complex issues: i) the RNA shedding level varies both between infected individuals and during the course of the disease for an infected individual [44]; ii) the concentration of particles of viral RNA in wastewater is affected by several extraneous factors including changes in dilution, ambient temperature, exposure to various chemicals and the collection method [45,46]; iii) measurements of RNA viral load can vary according to the RNA detection method used [[47], [48], [49]]. For these reasons, the ability to measure and monitor viral RNA loads in wastewater is a necessary but not sufficient condition for achieving improved accuracy for wastewater-based models [50].

A comprehensive analysis of wastewater data is further complicated by the fact that various contributory factors exhibit spatio-temporal variation. These include, but are not limited, to: i) catchment population - shedding profile, population mobility, vaccination status; ii) network properties - rainfall dilution affecting the quality of wastewater, pumping system, chemical status of the water; iii) sampling considerations - collection methods, processing methods, sampling frequency; iv) biochemical analysis - sample processing protocol [51]. Therefore, an essential step towards realising the full potential of wastewater data for disease monitoring is the development and validation of statistical methodology that incorporates these contributory factors and captures the spatio-temporal variation in wastewater data metrics. Here, we review several modelling approaches that use wastewater data as an epidemiological surveillance tool. In addition to a review of published literature, we describe a number of collaborative groupings whose outputs are unpublished but available online in the form of dashboard and open-source monitoring tools.

## 2 Methods

**Fig. 1:**
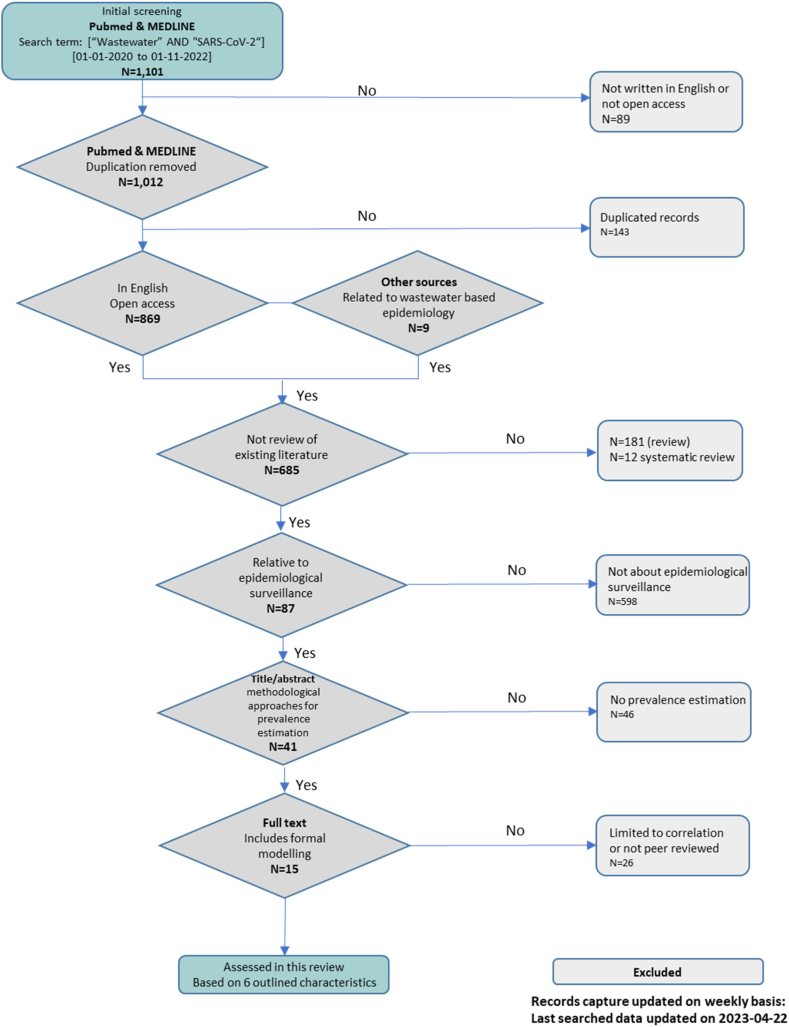
Selection criteria and search terms.

To identify articles for this review, we followed PRISMA guidelines focusing on open access publication in this area [52] for searching two main databases, PubMed and MEDLINE, supplemented by a list of relevant articles identified by the research team. All open access articles indexed in PubMed or MEDLINE databases between January 1, 2020 and April 22, 2023 including both main keywords (“SARS-CoV-2” AND “Wastewater”) in the title, abstract or main text (total of 1012 unique open access articles) were included. We also included 9 articles which did not have a direct index in the above databases. We then removed 143 duplicated records across the two databases and applied stage-wise selection criteria starting with 869 publications and 9 more from other sources, of which we excluded 181 literature reviews and 12 systematic reviews. The titles of all 685 remaining articles were screened for their relevance to the wastewater-based estimation of case prevalence in the population, resulting in 87 remaining articles of which 41 were selected based on our abstracts review (see Fig. 1 for details of selection criteria). We first summarised the 41 short-listed articles in detail; see Table S2 for a summary. We then characterised the 15 articles that met the following two criteria: i) they focused on wastewater epidemiology; ii) they used formal modelling approaches to predict COVID19 prevalence using viral measures obtained from wastewater that went beyond simple measures of association. (Supplementary Tables S1a and S1b for details of search strategy and hierarchy of selection).

Articles were imported and managed in Mendeley Version 1.19.8. To assess quality of articles, including study design, outcome measurement and bias, we used the guidelines of the Centers for Disease Control [53]. Although we only included studies whose main focus was on the development of predictive modelling approaches based on wastewater data, we also documented any emerging themes in the literature as an additional guide to the existing state of knowledge and development. Our final list includes serial reports from a single source such as national governments, each of which we counted only once in our list of distinct articles.

We further described and assessed these 15 articles in detail based on the following 6 characteristics.

● *Modelling approach:* which methods were used to analyse the data and create predictive models?
● *Temporal Coverage:* when did the data collection begin and end, and what was the time-interval used in the analysis, such as daily, weekly or a space-only analysis using aggregated data over time?
● *Spatial Coverage:* the spatial resolution at which wastewater viral concentrations are measured, e.g. the Waste Water Treatment Plant (WWTP) catchment level, and the resolution of the modelling output.
● *Sample Size:* Sample size and the type of sample used in each study directly impacts the statistical power and precision that can be achieved at any given spatial resolution. Also, the sample size required to achieve a given level of precision increases as the required spatial resolution is refined. We assessed each study based on number of samples that were included and how diverse the sampling locations were: from urban residential areas only, or including both urban and rural areas?
● *Wastewater sample origin:* WWTP or other sources such as sewer networks
● *Covariates:* which covariates were included in the model, and are they recorded as spatial-only, temporal-only or spatio-temporal measurements?

## 3 Results

We observed an evolving theme whereby the initial focus up to July 2020 was on the development and optimization of water sampling protocols, curation of collected wastewater sample and lab-based analysis of viral load for generation of daily measures [[54], [55], [56]] (see Table 1). Another aspect was the development of data visualisations through dashboards for real-time reporting across the globe; see Table 2 for a summary of the dashboards identified. Investigations around existing association between RNA viral load and number of infected COVID-19 cases started after July 2020 [[57], [58], [59]]. Development of formal modelling approaches that considered the use of wastewater data for prediction of COVID-19 cases in the community began since 2021 [[35], [36], [37], [38],^43^,[60], [61], [62], [63]].

**Table 1:** Summary of the 15 published WBE articles that included formal modelling and were accordingly selected for detailed review. Articles are presented in ascending date order). For details on all 38 screened articles including those which used only descriptive statistics to investigate correlation between wastewater-based measures and case numbers please see Supplementary Table S2.

No.	Authors	Temporal Coverage		Spatial Coverage		Sample size and diversity		Modelling approach	Covariates
		Start date	End date	Level	Country – City	Population and sites	Wastewater		
1	Krivoňáková et al., 2021 [60]	September 2020	March 2021	Single city	Slovak Republic - Bratislava & Petrzalka	Bratislava = 600,000 Petrzalka = 125,000 2 WWTP sites	Automatic sampler device: 50 mL samples every 15min for 24 h resulting in total of 50 analysed samples	Regression models: relationship of wastewater data and COVID-19 case count Time-series analysis: wastewater time series and various time lags of positive RT_qPCR test and COVID deaths. Applied a cross correlation function to identify the best match. GAM model used for illustration of the smoothed curves of time-series. Relationships examined: - WW and case counts - WW and +vs test counts - WW and COVID deaths	Viral particles
2	Srinivas et al., 2021 [61]	Not applicable	Not applicable	Multiple states	USA-13 states	Total population of USA (328.23 million) were divided into state based on age-bands	Auto-sampler installed at targeted locations identified by the fuzzy model	Serial connection network. Fuzzy-Bayesian optimization model Relationships examined: - WW and case counts	Urban, Rural demographics Migration rate (high/low) Quarantine facilities (good, pool) Comorbidities (i.e. Respiratory, CKD) Strict regulations (Yes, No) Education (poor, good) Healthcare facilities (good, pool) Temperature and weather conditions (below 10, 10–20, 20–30 and above 30) Population density (low, high) Population demography (male, female) Age-bands (<18, 19–29, 30–49, 50,84, 85+) Comorbidities (Respiratory, kidney, obesity, hypertension and none)
3	Omori et al., 2021 [62]	March 22, 2020	August 11, 2020	Single state	USA - Massachusetts	1 WWTP: with two major streams	3-7 times sampling per week	Maximizing the likelihood function considering a Poisson sampling process. Multiple regression analysis: to pin point heterogeneity between viral load and reported number of cases. Relationships examined: - WW and case counts	Concentration of a human fecal indicator Age group incidence Viral load
4	McMahan et al., 2021 [63]	May 27, 2020	August 25, 2020	Single city	USA –South Carolina	Three areas of Clemston town, total of ∼48,000 residents 3 sewersheds	Manual sampling – twice a week or weekly – 500 mL in plastic bottles	Monte Carlo simulation – SEIR model: susceptible, exposed, infectious, and recovered (model parameters: Median incubation period (fixed) = 0.2 = 5 days . Relationships examined: - WW and case counts	Virus mass rate Rainfall Assuming a 5 day of incubation (Wölfel et al., 2020) Model parameters: R0 = 1.5 | 2.0 | 2.5) R shiny app
5	Amereh et al., 2021 [35]	September 2020	April 2021	Single city	Iran – Tehran	6 medium-sized WWTPs 1 large WWTP site Urban Biweekly flow	Manual sampling in the 6 Auto sampling in the large plant site	Monte Carlo Simulation	Daily total number of SARS-CoV-2 RNA copies in wastewater Daily flow rate of wastewater Shedding rates
6	Galani et al., 2022 [36]	August 31, 2020	March 21, 2021	Regional	Greece - Attica region, includes Athens metropolitan area and suburbs.	1 wastewater site in Attica	Manual sampling in pre-cleaned HDPE 2 L bottles	Linear regression model of time-series data. Multi-layer artificial neural network (ANN) using backpropagation algorithm. Bayesian Distributed-Lag non-linear model of Poisson family with log-link (positive regression coefficient from gamma distribution with unknown shape and scale) Relationships examined: - WW and positive case counts - WW and hospital admission - WW and ICU admission	RNA viral load Fingerprint data (binary variable: 0 = if RNA copies in wastewater > limit of quantification, 1 = for inverse scenario) Number of positive cases Incubation period of SARS-CoV-2 (Wölfel et al., 2020) Viral Shedding in feces New hospitalisations Code and dashboard
7	Scottish Government [37]	December 2020	April 28, 2022	National	Scotland – UK no regional breakdowns	No details provided	No details provided	Two approaches for predicting R number: 1-Using wastewater derived data 2-Using data from an agent-based model Relationships examined: - Comparing R number derived from WW against that derived using ABM	Model outputs are reported but no information on covariates
8	Fitzgerald et al., 2021 [38]	March 1, 2020	Jan 31, 2021	National (50 % of Scotland)	Scotland – UK	28 WWTPs from 2.7 Million (50 % of 5.3 M Scottish population) for 917 m^2^ catchment (1.2 % of the total 77,933 m^2^)	Refrigerated Autosamplers: 1 sample per hour during the 24 h – composite 24-h samples used Data available from: https://informatics.sepa.org.uk/RNAmonitoring/	1 Linear regression for prediction of daily concentration from independent variables: catchment population and site 2 Spearmen's rank correlation between viral concentration and number of positive cases 3 Linear mixed model: with fixed coefficients for daily viral load and random intercept and coefficients for each catchment. Relationships examined: - WW and case counts	Catchment area (latitude, longitude) Population density Number of wastewater samples Deprivation index Access index as a measure for access to healthcare services
9	Proverbio et al., 2022 [39]	Open data sources from 2020	Up to August 2021	12 regional areas: Europe & North America	Multi-country in Europe	Open source data with variable locations and sampling methods	Not applicable	COVID-19 Wastewater Analyser (CowastewaterAn) Modified SEIR model with the Extended Kalman Filter.	Parameter based SEIR model: Initial size of E = exposed and I = infected are automatically computed. The average ratio of total is set to 3 initially and then to 1.8.
10	Kuhn et al., 2022 [40]	November 1, 2020	Ongoing	Single city: Oklahoma City	USA – Oklahoma City	13 locations	Autosamplers: 900 mL grab samples	Spatio-temporal model of total daily cases across sewersheds was automated using Esri's ModelBuilder. Spatial census areas from Esri's ArcMap and QGIS were used to quality control the overlayed polygon for sewershed areas. GLM models: viral load & incident COVID-19 cases (per 100,000). Relationships examined: - WW and incidence	Population size Ethnic composition (proportion of ethnic populations = continuous variable) Proportion of population aged 65 years or older (continuous variable) Median income (continuous variable) Day and month of the year (categorical variable)
11	Pájaro et al., 2022 [41]	May 2020	May 2021	Single City: Galicia with predictions being at local level for small and medium size municipalities	Galicia - Spain	11 WWTP Population between 2000 and 23,000	Autosamplers: 24 h composite sample. 1-2 samples per week	SEIR model: susceptible, exposed, infectious, and recovered. model parameters: constant recovery rate = 1/14 (cumulated incident for a 14 days time interval) Infection rate = estimated from public health data on infected individuals	Measurement of viral load in wastewater Cumulative incident rate per 14 days
12	Petros et al., 2022 [64]	Autumn 2020	Spring 2021	Single University site	Colorado Mesa University - USA	6 on-campus swage sites	Autosampler: 24 h composite sample	Multiple linear regression Relationships examined: - Combined measures from multiple sources - WW and case positivity	Demographics Contact tracing Wifi-based location data Pathogen surveillance from wastewater Diagnostic testing
13	Zhao et al., 2022 [32]	September 1, 2020	October 4, 2021	Multi regions	City of Detroit, and Wayne, Macomb, Oakland	1 wastewater authority in southeast Michigan (407 samples)	VIRADEL sampling method & 24-h composite sample	Autoregression models	RNA viral load
14	G. Li et al., 2023 [43]	June 1, 2021	March 30, 2022	Lower super output areas (LSOAs) in England	England- UK	303 Sewer Treatment Plants 32,844 LSOA	Autosampler: 24 h composite sample info provided in separate paper by [65]	Spatially continuous model using Bayesian modelling framework	Index of Multiple Deprivation Black and minority ethnic Population density Young pop percentage (<16) Older pop percentage (>75) Industrial fraction Genome coverage Single Nucleotide Polymorphism number Hyperparameters Residual variance Variance of regional random effect Variance of the temporal random effect Correlation range (km) in the Matern covariance Variance in the Matern covariance Temporal AR1 coefficient
15	Vaughan et al., 2023 [42]	Variable per region– information available at (https://sphere.waterpathogens.org/map)	Variable per region – information available at (https://sphere.waterpathogens.org/map)	Multi regions	108 Cities in Five Countries (Scotland, Catalonia, Ohio, the Netherlands, and Switzerland)	For each region information are available at (https://sphere.waterpathogens.org/map)	For each region information are available at (https://sphere.waterpathogens.org/map)	Machine Learning (Random Forest – 100 trees, 80 % training set and one step ahead prediction based on sampling frequency on each WWTP that is automatically detected from each dataset)	Sampling frequency Flow rate Viral incubation period Viral loads

**Table 2:** Links to online dashboards relating to wastewater data.

Country		Title	Dashboard link
**United Kingdom**	England	EMHP:viral load concentration over time for different regions	(https://wastewaterw.gov.uk/government/publications/monitoring-of-sars-cov-2-rna-in-england-wastewater-monthly-statistics-1-june-to-1-november-2021/emhp-wastewater-monitoring-of-sars-cov-2-in-england-1-june-to-1-november-2021)
		Wastewater testing coverage data	(https://wastewaterw.gov.uk/government/publications/wastewater-testing-coverage-data-for-19-may-2021-emhp-programme/wastewater-testing-coverage-data-for-the-environmental-monitoring-for-health-protection-emhp-programme)
	Wales	Wastewater Monitoring in Wales	https://gov.wales/sites/default/files/publications/2022-06/wastewater-monitoring-23-june-2022.pdf
	Scotland	Modelling the epidemic	https://wastewaterw.gov.scot/publications/coronavirus-covid-19-modelling-epidemic-issue-no-97/
		Scottish Environment Protection Agency	https://informatics.sepa.org.uk/RNAmonitoring/
**United States**	Global	John Hopkins University if Medicine | Coronavirus resource center	https://coronavirus.jhu.edu/map.html
	Country wide	Centers for Disease Control and Prevention	https://covid.cdc.gov/covid-data-tracker/#wastewater-surveillance
	Utah	Utah Department of Environmental quality – Water Quality	https://deq.utah.gov/water-quality/sars-cov-2-sewage-monitoring
	Tempe- Arizona	Tempe-Wastewater Dashboard	https://covid19.tempe.gov/#Explore
	South Caroline	COVID-19 Wastewater Model (McMahan et al., 2021)	https://github.com/scwatson812/COVID19WastewaterModel https://rennertl.shinyapps.io/Wastewater_projections/
	North Carolina	COVID-19 Wastewater Dashboard	https://wastewater.covid19.mathematica.org/
		NCDHHS COVID-19 Response	https://covid19.ncdhhs.gov/dashboard/data-behind-dashboards
	California State (Stanford University tool)	Sewer Coronavirus Alert Network (SCAN) tracking	https://soe-wbe-pilot.wl.r.appspot.com/charts#page=overview
**New Zealand**	New Zealand	Wastewater Surveillance	https://esr-cri.shinyapps.io/wastewater/#region=Wellington&log_or_linear=log&period=allTimeButton

### 3.1 Modelling approaches

We categorised the modelling approaches into five themes.

I) linear regression and time series analysis: this category includes studies that use the wastewater measures collected over time to derive the relationships between COVID-19 case numbers and the SARS-CoV-2 Viral load in wastewater [36,38,60,62,64].
II) Compartmental SEIR models and agent-based models: this category includes studies that used compartmental Susceptible-exposed-infectious-recovered (SEIR) approaches as traditionally used or in a more sophisticated form as in Proverbio et al. (2022) or approaches where each element is estimated through simulation of stochastic processes such as estimation of disease prevalence through Monte Carlo simulation [35,39,41,63,66].
III) Machine learning models: This category includes a single study by Vaughan et al., 2023 which used a machine learning approach
IV) Bayesian models: this category includes a single study by Srinivas et al., 2021 which developed a Bayesian model approach.
V) spatio-temporal models: this category includes the models which specifically incorporated both spatial and temporal data into their prediction models and were able to provide estimates of viral load or COVID-19 prevalence at refined special resolutions [37,40,43] (see Table S3 for categorisation and type of modelling conducted in each study). We now summarise each of the 15 papers in greater details on each one of the above criteria.

Krivoňáková et al. (2021) conducted their analysis on a regional level from two major WWTPs, using a time series modelling approach originally on daily data, then moving to weekly time series. They treated samples for each city separately in their models. One of the characteristics of the Krivoňáková et al. approach was their ability to minimize the size of wastewater sample required for detecting a SARS-CoV-2 case through a positive RT-qPCR signal in wastewater. They reported achieved detection limits of 1 positive RT-PCR case per 4808 and 1 per 8099 inhabitants for the two cities considered. They used a linear regression model with weekly numbers of deaths in the city of Bratislava and Petrzalka, Slovakia, as the response and the log-transformed weekly averaged viral particle counts from two WWTPs lagged by four weeks as the explanatory variable, obtaining a coefficient determination value (R^2^) of approximately 0.8.

Srinivas et al. (2021) did not create an explicit calibration between wastewater measures and clinical cases. Rather, they developed a Bayesian network model aiming to locate wastewater sites that had a high probability of indicating a local outbreak. They used 11 factors including demographic variables, migration rates, healthcare and educational facilities as well as general population measures such as age and comorbidities including: respiratory, kidney, cardiovascular, obesity, liver, diabetes and hypertension conditions. The structure of their model was informed by applying fuzzy logic to the judgements of a group of experts. While assessment and characterisation of this specific study did not completely fit the five defined categories of our study, we considered the approach to be relevant both from the design angle and through its incorporation of comorbidity factors.

Omori et al. (2021) investigated the lag between time series of case numbers within different age groups and wastewater viral load. They showed that a rise in incidence can be detected up to 10 days earlier using wastewater viral load than by reported case numbers.

McMahan et al. (2021) modelled the time series of wastewater data from three WWTPs that covered the city of Clemson, South Carolina, USA by linking a classical SEIR model of case-prevalence with a non-linear deterministic model for the time-varying SARS-CoV-2 RNA load in wastewater. This modelling embeds an approximation of the shedding profile of an infected individual within the SEIR model to predict COVID case numbers, which were then validated against the observed clinical case numbers. For surveillance purpose, they also proposed a simple calibration criterion for the relationship between viral load per litre in wastewater and the number of incident cases per day.

Fitzgerald et al. (2021) investigated associations of viral load and daily case numbers at the catchment area level in Scotland, using Spearman correlation followed by a series of univariate linear regression models and then the development of a linear mixed model with random intercepts and slopes specified at the catchment area level.

Galani et al. (2022) proposed a Bayesian non-linear Poisson distributed-lag model that uses wastewater-based measurements to predict the number of confirmed positive COVID-19 cases as well as COVID hospitalisation and ICU admissions. Their modelling suggested that viral load in wastewater leads the above three clinical indicators by between 2 and 8 days.

On March 10, 2022, following the work of Fitzgerald et al. (2021), the Scottish government published initial figures comparing trends of average viral load in wastewater against case positivity rates from the CIS survey covering the period May 14, 2020 to March 4, 2022 [66,67]. Viral load estimates were made for the 32 local authorities (LAs) of Scotland and were updated continuously with the development of formal modelling at the national level [37].

Kuhn et al. (2022) analysed wastewater data from 13 locations in Oklahoma City, USA. They used Poisson log-linear models fitted separately to wastewater from each location and to all 13 locations combined, but did not attempt to fit an explicitly spatio-temporal model. They considered a range of socio-demographic factors at the sewershed catchment area level, including proportions of the population in different ethnic groups, proportion of the population aged 65 or more, mean income and numbers of notified COVID-19 cases. Their modelling showed a lead time of between 4 and 10 days in detecting COVID-19 outbreaks.

Proverbio et al. (2022) developed a more generalized pipeline, the “COVID-19 Wastewater Analyser,” which incorporates a range of pre-validated parameters such as infectivity rate and population size, as well as transition rates from Exposed status to Infected status (E to I) and from Infectious to Removed status (I to R) [68] within an SEIR model. They then used their model to estimate case numbers and epidemic indicators such as the reproduction number [39].

Pájaro et al. (2022) focused on predicting the evolution of the pandemic with a stochastic Susceptible-Infected-Recovered (SIR) model that was validated using data of small and medium size municipalities with total populations of 2000 to 23,000 individuals from Galicia, Spain. The SIR model was developed using a Chemical Master Equation (CME) as the base for incorporating the stochasticity of chemical status. The group used a Stochastic Simulation Algorithm and considered the number of clinically positive cases as one realisation of the simulation.

Petros et al. (2022) developed a linear regression model using the Mesa University-USA campus data. Their model incorporates uniquely a set of widely accessible measures such as demography, contact tracing, wifi-based location data, viral load and COVID testing data.

Zhao et al. (2022) developed four statistical modelling approach: liner regression, autoregressive integrated moving average (ARIMA), seasonal ARIMA, vector autoregressive model using wastewater samples from 407 WWTPs over a 12 month period. They have shown that seasonal ARIMA and vector autoregressive models had an optimized prediction for COVID-19 cases.

Li et al. (2023) developed a Bayesian geostatistical model that uses weekly measurements of wastewater viral concentration at the 303 sewage treatment works across England to predict wastewater viral concentration for all the 32,844 lower super output areas in the country. To help prediction, this model incorporated a range of covariates including index of multiple deprivation, ethnicity proportion based on census 2011, land cover, age structure and wastewater genomic data.

Vaughan et al. (2023) investigated the challenges associated with machine learning approaches for obtaining early detection signals from wastewater samples. They used data from 108 cities from 5 countries.

### 3.2 Temporal Coverage

The majority of studies published early in 2021 used the data available from the start of the pandemic, with less than a year of temporal coverage. Among the 15 studies that we characterised in detail, Kuhn et al. (53) benefited from 20 months of data coverage [40], followed by a 17 month coverage for models developed by the Scottish government [66]. Notably, both Kuhn et al. (2022) and the Scottish government report [66] developed their approaches using a pipeline that benefits from near-real-time data. Vaughan et al. (2023) used openly available data from (https://sphere.waterpathogens.org/map) with variable coverage range. The other 10 studies used less than one year's data and we found no indication of regular data updates [35,36,38,39,41,[60], [61], [62], [63], [64]].

Temporal resolution of the data was at daily level for all of these studies. Similarly, all developed model outputs for predicting case numbers were on a daily scale across different studies.

### 3.3 Spatial Coverage

We distinguish between the spatial resolution of input data at which the WW measurements are made and the resolution of the modelling outputs. The spatial resolution of input data across five different projects [[35], [36], [37], [38],^62^] was at the WWTP catchment level. COVID incidence, prevalence and hospitalisation were predicted at a spatial resolution that does not match directly the spatial resolution of the input data. Models were constructed to produce outputs at a smaller area level and then converted to the regional, site or city level for reporting. Of all the reviewed models, we found one model developed by Li et al. (2023) with explicit consideration for spatial prediction of viral load in the refined geographical areas while the rest of models were focused on the spatial granularity provided by source data with no explicit refinements on spatial predictions.

Srinivas et al. (2022) and Proverbio et al. (2022) used datasets spanning multiple states and multiple countries to validate their models. However, all their predictions were at country or state level. Six research groups [35,40,41,60,62,63] developed modelling approaches using a single city's data.

### 3.4 Sample size & Wastewater sample details

Eight of the 15 studies that we reviewed in detail used an auto-sampler from wastewater treatment plants [32,35,40,41,43,60,61,64]. Only two of these gave details of the number of sampling rounds and the number of samples per round: Krivoňáková et al. (2021) reported that they used more than 50 samples; Amereh et al. (2021) reported results from 13 rounds of sampling over approximately six months. The studies by McMahan et al. (2021) and Galani et al. (2022) used a manual sampling approach. Omori et al. (2021) sampled each of the two main wastewater streams between three and seven times per week using an unstated method.

Limit of detection or quantification (LOD or LOQ) is a factor arising from the sampling process. This factor was considered in studies conducted by Krivoňáková et al. (2021), Galani et al. (2022) and Amereh et al. (2021) [35], [36], [60]. Krivoňáková et al. state their detection limit for each region at a single date while noting that LOD and LOQ measures are defined based on total number of positive reported RT-qPCR test and therefore are highly dependent on the testing capacity of each region. Amereh et al. and Galani et al. use LOD and LOQ to define a threshold for case detection while Galani et al. found this to result in measures with insufficient sensitivity.

### 3.5 Outcome and covariates

Across all the studies, a range of epidemiological COVID outcomes have been considered, including number of reported cases (prevalence) or number of new cases (incidence), hospitalisation and death. None of the studies directly reports outputs at the exact level of the WW measurements, but some studies have addressed the problem of spatial misalignment between WW input and health outcome data. For example, McMahan et al. (2021) and Amereh et al. (2021) matched the WW data to the sewershed catchment area level data to estimate under-reporting rates; this provides a measure of the accuracy of disease burden; specifically when using WW as an early warning system, knowledge about accuracy of estimates contributes directly to effective allocation of lockdown or other measures and resources.

Four studies took the approach of predicting the population prevalence using the viral load measurement in wastewater as a covariate [35,36,60,62] used the Viral Mass rate as a covariate; this is very similar to viral load but is less seriously affected by dilution (McMahan et al., 2021). Srinivas et al. (2021) used a range of covariates in their Bayesian model, including: demographic and socio-economic variables, such as age, gender, ethnicity and population density; WWTP covariates such as rainfall at the time of sampling; and shedding rate.

The SEIR models developed by McMahan et al. (2021) and Proverbio et al. (2022) also used pandemic-related measures such as median incubation period.

## 4 Discussion

In this review, we have summarised a variety of wastewater-based surveillance models developed for studying the evolution of COVID-19 across diverse geographical regions. Our review has revealed that while the potential utility of wastewater resources is widely recognized, and global efforts to harness this potential are underway, only a limited number of studies have explicitly explored the spatio-temporal relationship between wastewater viral contents and prevalence of COVID-19 infections. Wade et al. (2022) have documented the associated challenges, while Nicholson et al. (2021) have explicitly investigated the complexities of achieving an unbiased estimate of prevalence. The well-established advantages of wastewater sampling over direct sampling of at-risk populations are noteworthy. Firstly, wastewater sampling is more cost-effective than community testing. Secondly, it represents a non-intrusive approach. Thirdly, it is virtually immune to the selection biases that can arise with direct epidemiological measurements. Lastly, it can offer early detection signals to identify regional outbreaks. Carducci et al. (2020) and Kirby et al. (2021) have suggested a lead time of 5–10 days in the wastewater viral load before the onset of a regional outbreak that would be identified by clinical testing [[69], [70]].

To fully realise the potential of wastewater data for COVID monitoring, several challenges persist. These encompass the need to account for variability in the characteristics of wastewater treatment plant sites, the selection of metric and the choice of sampling methodologies. For instance, Peccia et al. (2020) demonstrated the presence of N1 and N2 gene targets of SARS-CoV-2 in the solid component of wastewater, known as sludge. However, none of the model-based approaches that we identified have utilized a sludge samples. Shedding profiles, rainwater dilution and treatment plant variability have been identified as some of the relevant factors, as discussed by Petala et al. (2022), Wang et al. (2020), Zhu et al. (2021a) and Morvan et al. (2021). These factors need to be appropriately incorporated in the modelling of wastewater data. The utilization of RNA viral load as a proxy for disease prevalence in wastewater-based surveillance presents intricate challenges. Variability in RNA shedding levels among infected individuals and over the course of infection [44], as well as the influence of external factors like dilution, temperature, chemical exposure, and detection methods, emphasize that measuring viral RNA loads in wastewater, though necessary, is insufficient for achieving improved model accuracy [[45], [46], [47],^51^]. Additionally, a comprehensive analysis of wastewater data is complicated by spatio-temporal variations in various contributing factors, such as population characteristics, network properties, sampling considerations, and biochemical analysis protocols. To fully unlock the potential of wastewater data for disease monitoring, there is a pressing need for the development and validation of statistical methodologies capable of accounting for these factors and capturing the nuanced spatio-temporal variations in wastewater metrics. In this review, we explore various modeling approaches that leverage wastewater data as a valuable epidemiological surveillance tool, drawing insights from both published literature and collaborative groups that have made their findings accessible through dashboards and open-source monitoring tools. These efforts collectively address the multifaceted challenges inherent in wastewater-based surveillance, not only for COVID-19 but also for broader public health applications.

Furthermore, the harmonisation of sampling and measurement protocols holds promise for enhancing the comparability and reproducibility of findings from wastewater studies (Kasprzyk-Hordern et al., 2022). Our review has revealed considerations across all studies for various contributing factors in the multi-dimensional nature of the wastewater sampling and testing process, as discussed in Olesen et al. (2021) and Zhao et al. (2022). Nevertheless, adopting a comprehensive framework that accounts for sampling, testing and reporting variables in a spatio-temporal manner would bring us closer to a harmonised approach.

Since the start of the pandemic, many groups have worked on wastewater-based surveillance methods, using various ways of communicating their findings to diverse groups of stakeholders, describing logistical challenges for WBE in Africa, the Netherlands, Turkey and England [71]. Takeda et al. (2021) [72] described the development and challenges in Indonesia, Japan and Vietnam and Wade et al. (2022) discussed lessons learnt from the UK. A dashboard of daily frequencies seems to be the most common approach for real-time documentation and communication of the state of the epidemic; see Table 2. In the UK, cross-country programmes led by the UK Health Security agency have generated various dashboards and reports. In England, Environmental Monitoring for Health Protection (EMHP), has published weekly reports [73] providing a seven-day rolling average of virus concentration (gene copies per litre) in 303 treatment plant sites as well as open source data [74]; see Supplementary Fig. S1 for map of WWTPs across England. The Scottish government produced a weekly report (https://www.gov.scot/publications/coronavirus-covid-19-modelling-epidemic-issue-no-97/) of progress with the wastewater monitoring program [66]. In Wales, the first report (https://gov.wales/sites/default/files/publications/2022-06/wastewater-monitoring-23-june-2022.pdf) of the wastewater monitoring program was released on June 23, 2022 [75]. In the USA, 43 health departments were funded to provide data on SARS-CoV-2. Here, we summarise the identified dashboards across states. The COVID Data Tracker dashboard (https://covid.cdc.gov/covid-data-tracker/#wastewater-surveillance) of SARS-CoV-2 RNA levels, developed by the Centers for Disease Control and Prevention (CDC), illustrates for 407 treatment plant sites across the United States the 15-day detection proportions across the country (CDC, 2020a). The dashboard (https://deq.utah.gov/water-quality/sars-cov-2-sewage-monitoring) of the SARS-CoV-2 sewage monitoring project run by Utah Department of Environmental Quality illustrates insights from 42 treatment plants (80 % of state's population) and shows: for **wastewater,** in each plant the trends of overall viral load as gene copies in sewage per person per day; for **community**, the total numbers of new daily cases per 100,000 population per day [76]. Another example is from the city of Tempe in Arizona state, whose dashboard (https://covid19.tempe.gov/#Explore) illustrates: for **wastewater**, trends of viral load as weekly average of gene copies per litre of wastewater; for **community**, total number of COVID-19 positive test results per 100,000 per zip code; for **demographics**, area statistics by age group, sex, ethnicity and household ownership status [77]. The North Carolina dashboard (https://wastewater.covid19.mathematica.org/) provides: for **wastewater**, trends of viral load as flow normalised viral load (MGC/person/day); for **community**, summaries of cases and deaths with a 7-day rolling average of new cases [78]. Research from this state led by Barua et al. (2021) [79] also used the published daily incidents from data behind the dashboard available at: https://covid19.ncdhhs.gov/dashboard/data-behind-dashboards.

It is essential to acknowledge a notable limitation within the scope of this study. Despite our comprehensive analysis of 15 selected articles, we did not identify a substantial body of research that explicitly stratified wastewater data based on variants of concern or vaccination status. The absence of such stratification is a limitation as it prevents us from fully understanding the impact of emerging variants and vaccination efforts on viral load dynamics in wastewater. Given the evolving nature of the COVID-19 pandemic, these factors play a crucial role in shaping disease dynamics and warrant further investigation. Future studies that incorporate stratification based on variants of concern and vaccination status are needed to provide a more comprehensive and nuanced understanding of how these factors influence the interpretation of wastewater-based surveillance data. Such research could offer valuable insights into the effectiveness of vaccination campaigns and the prevalence of specific viral variants within communities, ultimately enhancing the utility of wastewater surveillance as a public health tool.

**Fig. 2:**
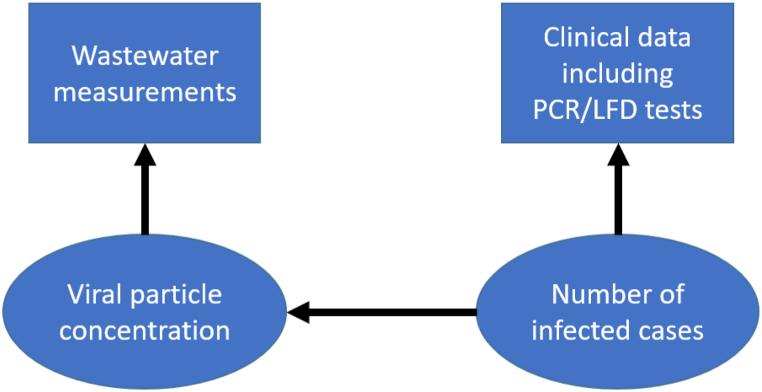
A conceptual model for relating wastewater and infection data (rectangles) to latent viral particle concentration and infection risk (ellipses).

Many countries have initiated surveillance approaches based on wastewater data. However, our review, up to April 2023, suggests that three important statistical issues have not been addressed adequately. Firstly, while several studies demonstrated spatial and temporal variability in the RNA viral load, most have focused on spatially aggregated temporal changes. Secondly, only one study has developed a spatio-temporal model using wastewater data collected at sewage treatment plants to predict viral loads at fine spatio-temporal resolution across an entire country. Thirdly, most existing work either considers wastewater data in isolation, or relates it to COVID-19 infection rates as an explanatory variable within a regression modelling framework, with little consideration of the fact that wastewater viral load and clinical outcomes such as infection rates are observed with error. Instead, we believe that the spatio-temporal variation in viral concentration and risk of infection should be modelled jointly as a latent process whose evolving state is informed by observations of wastewater RNA measurements and clinical data such as PCR or LFD test data; see Fig. 2. In the development of any model-based approaches an optimized and quality-controlled reporting pipeline to deliver data at regular, ideally daily, intervals into a suitably structured and spatially resolved database would facilitate a near-real-time reporting process.

In conclusion, our review has shown that the global effort in harnessing wastewater data for its known potential in providing an efficient and timely surveillance tool is evolving rapidly. However, there remains a need for more research to develop an explicitly spatio-temporal wastewater-based surveillance system that can be validated for use in a range of public health settings.

## Funding

This work was funded by The Department for Health and Social Care (Grant ref: 2020/045) with additional support from The 10.13039/100012338Alan Turing Institute (EP/W037211/1) and in-kind support from 10.13039/100012100The Royal Statistical Society.

## Data availability statement

The data associated with this study has not been deposited into a publicly available repository. For the reproducibility of our search approach, the data about our search is included in the supplementary material of this article and all research pieces are referenced. No other data has been used as part of this review.

## CRediT authorship contribution statement

**Fatemeh Torabi:** Writing – review & editing, Writing – original draft, Visualization, Formal analysis, Data curation, Conceptualization. **Guangquan Li:** Writing – review & editing, Writing – original draft, Investigation. **Callum Mole:** Writing – review & editing. **George Nicholson:** Writing – review & editing, Methodology. **Barry Rowlingson:** Writing – review & editing. **Camila Rangel Smith:** Writing – review & editing. **Radka Jersakova:** Writing – review & editing. **Peter J. Diggle:** Writing – review & editing, Writing – original draft, Supervision, Conceptualization. **Marta Blangiardo:** Writing – review & editing, Writing – original draft, Supervision, Conceptualization.

## Declaration of competing interest

Authors have declared no competing interest with this work.
