# Supplementary material for: Wastewater-based surveillance models for COVID-19: A focused review on spatio-temporal models
Source: Heliyon. 2023 Nov 8;9(11):e21734. doi: 10.1016/j.heliyon.2023.e21734 (PMC10694161; doi:10.1016/j.heliyon.2023.e21734)
Supplement: Multimedia component 1 [file mmc1.docx]

**Wastewater-based surveillance models for COVID-19: a focused review on spatio-temporal models**

Fatemeh Torabi^1, 2^, Guangquan Li ^1,3^, Callum Mole^1,4^, George Nicholson ^1,5^, Barry Rowlingson ^1,3^, Camila Rangel Smith ^1,4^, Radka Jersakova ^1,4^, Peter J Diggle^1,6*^, Marta Blangiardo^1, 7*^

1. Turing-RSS Health Data Lab, London, UK
2. Population Data Science HDRUK-Wales, Medical School, Swansea University, Wales, UK
3. Applied Statistics Research Group, Department of Mathematics, Physics and Electrical Engineering, Northumbria University, Newcastle upon Tyne NE1 8ST, UK
4. The Alan Turing Institute, London, UK
5. University of Oxford, Oxford, UK
6. CHICAS, Lancaster Medical School, Lancaster University, England, UK
7. MRC Centre for Environment and Health, Department of Epidemiology and Biostatistics, Imperial College, London, UK.

# **Supplementary materials**

Figure S1 – Wastewater sampling sites across England as reported by [EMHP](https://www.gov.uk/government/publications/monitoring-of-sars-cov-2-rna-in-england-wastewater-monthly-statistics-1-june-to-1-november-2021/emhp-wastewater-monitoring-of-sars-cov-2-in-england-1-june-to-1-november-2021)


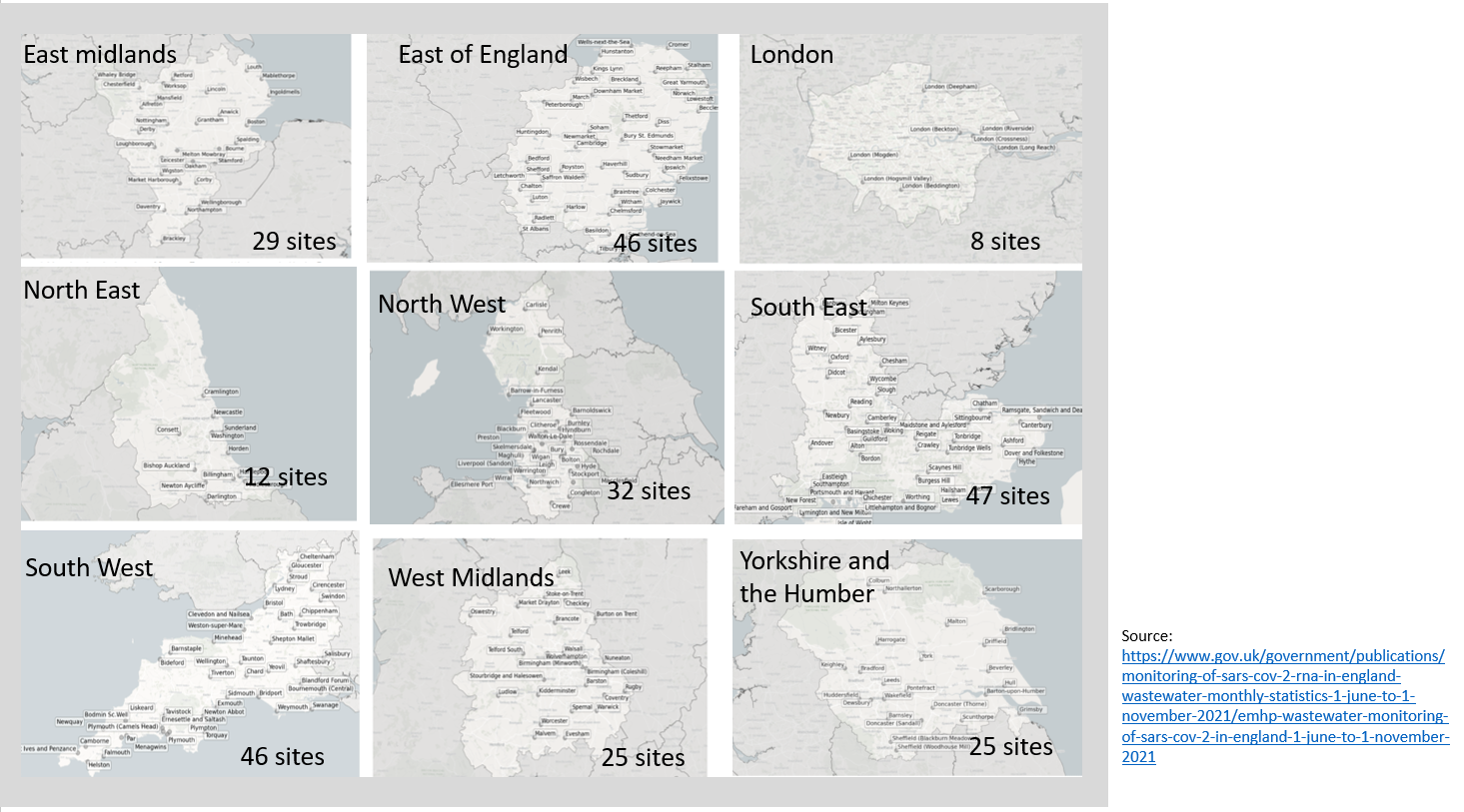


Table S1a – Hierarchy of PubMed search and total number of articles identified in each stage

| Search number | Query | Sort By | Filters | Search Details | Results |
| --- | --- | --- | --- | --- | --- |
| 1 | ("SARS-CoV-2"[All Fields] AND "Wastewater"[All Fields]) | Publication Date | from 2020 - 2020 | ("SARS-CoV-2"[All Fields] AND "Wastewater"[All Fields]) AND ((ffrft[Filter]) AND (2020:2020[pdat])) | 156 |
| 2 | ("SARS-CoV-2"[All Fields] AND "Wastewater"[All Fields]) | Publication Date | from 2021 - 2021 | ("SARS-CoV-2"[All Fields] AND "Wastewater"[All Fields]) AND ((ffrft[Filter]) AND (2021:2021[pdat])) | 455 |
| 3 | ("SARS-CoV-2"[All Fields] AND "Wastewater"[All Fields]) | Publication Date | from 2022 - 2022 | ("SARS-CoV-2"[All Fields] AND "Wastewater"[All Fields]) AND ((ffrft[Filter]) AND (2022:2022[pdat])) | 454 |
| 4 | ("SARS-CoV-2"[All Fields] AND "Wastewater"[All Fields]) | Publication Date | from 2023 - 2023 | ("SARS-CoV-2"[All Fields] AND "Wastewater"[All Fields]) AND ((ffrft[Filter]) AND (2022:2022[pdat])) | 152 |
| 4 | ("SARS-CoV-2"[All Fields] AND "Wastewater"[All Fields]) | Publication Date | 2020-2023 | "SARS-CoV-2"[All Fields] AND "Wastewater"[All Fields]  Duplications removed | 1101 |
| 5 | ("SARS-CoV-2"[All Fields] AND "Wastewater"[All Fields]) | Publication Date | Free full text | ("SARS-CoV-2"[All Fields] AND "Wastewater"[All Fields]) AND (ffrft[Filter]) | 1012 |
| 6 | ("SARS-CoV-2"[All Fields] AND "Wastewater"[All Fields]) | Publication Date | Free full text, Review | ("SARS-CoV-2"[All Fields] AND "Wastewater"[All Fields]) AND ((ffrft[Filter]) AND (review[Filter])) | 181 |
| 7 | ("SARS-CoV-2"[All Fields] AND "Wastewater"[All Fields]) | Publication Date | Free full text, Systematic Review | ("SARS-CoV-2"[All Fields] AND "Wastewater"[All Fields]) AND ((ffrft[Filter]) AND (systematicreview[Filter])) | 12 |

Table S2- All 39 selected articles – ordered by lead author last name, the modelling articles are blue highlighted.

| **No.** | **Authors** | **Temporal Coverage** | **Spatial Coverage** | **Country – City** | **Sample size and diversity** | **Wastewater sample details** | **Modelling approach** | **Covariates** |
| --- | --- | --- | --- | --- | --- | --- | --- | --- |
| 1 | (Amereh et al., 2021) | September 2020 until April 2021  Biweekly flow | Single city (partial coverage):  64% coverage of the capital city – serving 5+ Million individuals | Iran – Tehran | 6 medium-sized WWTPs  1 large WWTP site  Urban | Manual sampling in the 6  Auto sampling in the large plant site | Monte Carlo Simulation | Daily total number of SARS-CoV-2 RNA copies in wastewater  Shedding rates |
| 2 | (Barua et al., 2021) | 24 June 2020 (every Wednesday for 6 months) | Single city (partial coverage): Charlotte region of North Carolina | USA – North Carolina | 4 WWTPs in Charlotte and North Carolina | Manual sampling in sterile 1 L Nalgene bottles. | Spearman’s rank correlation to determine any correlation between RNA viral load and incident clinical cases. | Incident was calculated as difference between cumulative cases in two days:  [Government reported daily number of cases](https://github.com/wraldata/nc-covid-data/tree/master/zip_level_data/time_series_data/csv) |
| 3 | (Bibby et al., 2021) | Establishing framework of lead time of wastewater based prediction | | | | | | |
| 4 | (Bivins et al., 2020) | [https://wastewaterw.covid19wbec.org/](https://www.covid19wbec.org/) as a collaborative hub on WBE | | | | | | |
| 5 | (Faleye et al., 2021) | 18 June 2020 to 1 October 2020 (4.5 months) | Single County: Maricopa County, Arizona, USA | USA – Maricopa County, Arizona, 4South-west USA | 13 sites in Maricopa County - Arizona | Auto-sampler: forty-eight 24h composite samples | No prediction of population prevalence | --- |
| 6 | (Fitzgerald et al., 2021) | 1^st^ March 2020 to 31^st^ Jan 2021 | 28 sites from 2.7 Million (50% of 5.3M Scottish population) for 917 m^2^ catchment (1.2% of the total 77,933 m^2^) | National (50% of Scotland) | Scotland – UK | 28 WWTPs  Refrigerated Autosamplers: 1 sample per hour during the 24 hours – composite 24-h samples used  Data available from: https://informatics.sepa.org.uk/RNAmonitoring/ | Linear regression for prediction of daily concentration from independent variables:catchment population and site  Spearmen’s rank correlation between viral concentration and number of positive cases  Linear mixed model: with fixed coefficients for daily viral load and random intercept and coefficients for each catchment. | Site slope  Population density  Number of ww samples  Latitude  Longitude  Deprivation  Access indices (relative deprivation and access to healthcare services of a datazone) |
| 7 | (Galani et al., 2022) | August 31 2020 to March 21 2021 (6.5+ months) | Regional: Attica region of Greece, includes Athens metropolitan area and suburbs. | Greece - Attica | 1 site in Attica | Manual sampling in pre-cleaned HDPE 2 L bottles | They took 3 approaches:  1-Linear regression model of time-series data.  2-A multi-layer artificial neural network (ANN) using backpropagation algorithm.  3-Bayesian Distributed-Lag nonelinear model of Poisson family with log-link (positive regression coefficient from gamma distribution with unknown shape and scale) | RNA viral load  Fingerprint data (binary variable: 0=if RNA copies in wastewater > limit of quantification, 1=for inverse scenario)  Number of positive cases  Incubation period of SARS-CoV-2 (Wölfel et al., 2020)  Viral Shedding in feces  New hospitalisations  [Code and dashboard](http://trams.chem.uoa.gr/covid-19/) |
| 8 | (Giraud-Billoud et al., 2021) | July 22, 2020 to November 30, 2020 (~4 months) | Regional: Mendoza region integrated by 6 geographical regions of Argentina, with population of ~1.4 M | Argentina – Mendoza province | 2 WWTPs: E1 Paramillo & Campo Espejo | Manual sampling – no rainfall or storm events occurred during sampling collection period | No formal modelling just crude comparison of Viral load vs Weekly number of Cases. | --- |
| 9 | (gov.scot, 2022a) | Data up to 28^th^ April 2022 | National: Scotland – no regional breakdowns | Scotland – UK | No extensive details provided in the report | No details in the report | Two approaches for predicting R number:  1-Using wastewater derived data  2-Using data from an agent-based model | Model outputs are reported but no information on covariates |
| 10 | (Herrera-Uribe et al., 2022) |  | Single city: Muharraq Island (pop=260,000) | Kingdom of Bahrain (pop= ~1.5 M) | 65 composite sewage samples collected (minimum of 2 samples per month in 2020 – and 3 samples per month in 2021 with 6 samples from quarantine facilities ) | Auto-samplers  Processed in an ISO17025 accredited lab | Spearman-rank correlation  Mann-Whitney non-parametric test | Took measurements to account for shedding rate and viral concentration (dilution) into the account. |
| 11 | (Hemalatha et al., 2021) | 8 July 2020 to 6 August 2020 (<1 month) | Single city: Hyderabad –population of ~10 M | Hyderabad - India | 30 samples from multiple sources: WWTPs, equalization tanks, secondary clarifier tanks, gated community tanks | Manual sampling in 1 L plastic bottle. | 2 methods for calculation of cases in population: method 1 from (Ahmed et al., 2020) and method 2 from (Hellmér et al., 2014) | “Faeces excreted/person/day = 128 g. (Rose et al., 2015). One positive person sheds 107 RNA copies/g of faeces (maximum estimate)”  “Number of RNA copies excreted per mL of faeces = 107 . Volume of faeces excreted=120 mL (calculated by considering thedensity of human faeces is 1.07 g/mL (Foladori et al., 2020)” |
| 12 | (Juel et al., 2021) | October 2020 to March 2021 (6 months) | Site level viral analysis: 37 sites | Not mentioned in the manuscript but it’s from the same team as Barua et al. (Charlotte, North Carolina) | 37 sites and total of 53 samples | Autosampler: : 3 times a week – sampling 20mL every 20 min over 24h to derive a composite sample | No model for estimation of population prevalence  Anova, t-test and regression was used for sample analysis | --- |
| 13 | (Kopperi et al., 2021) | 5^th^ and 6^th^ December 2020 &  11^th^ December 2020 | Single city: Hyderabad –population of ~10 M | Hyderabad - India | Hourly samples for method 1 and daily sample for method2 | Two sampling approaches: method1-grab and method2-composite sample. | No formal modelling  Individuals in active phase of infection were crudely calculated based on:  Total number of individuals in the selected area/(35 days window period)/(14 days infection period) | --- |
| 14 | (Krivoňáková et al., 2021) | --- | Single city: Bratislava – population of ~ 0.6 M | Slovak Republic - Bratislava | 2 WWTPs sites in the Bratislava and Petrzalka | Automatic sampler device: 50 ml samples every 15min for 24 h | **Predicting positive PCR or deaths by**  Time-series analysis: wastewater time series and various time lags of positive RT_qPCR test and COVID Deaths.  Applied a cross correlation function to identify the best match.  GAM model used for illustration of the smoothed curves of time-series. | Viral load |
| 15 | (Kuhn et al., 2022) | 1 November 2020 – ongoing | Single city: Oklahoma City | USA – Oklahoma City | 13 locations | Autosamplers: 900 mL grab samples | Spatio-temporal model of total daily cases across sewersheds was automated using Esri’s ModelBuilder. Spatial census areas from Esri’s ArcMap and QGIS were used to quality control the overlayed polygon for sewershed areas.  GLM models: viral load & incident COVID-19 cases (per 100,000). | Population size  **Demographics including:**  Ethnic composition (proportion of ethnic populations=continuous variable)  Proportion of population aged 65 years or older (continuous variable)  Median income (continuous variable)  Day and month of the year (categorical variable) |
| 16 | (Kumar et al., 2022) |  | WBE-based reported temporal variations globally | | | | | |
| 17 | (McMahan et al., 2021) | 27 May 2020 to 25 August 2020 (3 months) | Single city: Three areas of Clemston town, total of ~48,000 residents | USA –South Carolina | 3 sewersheds | Manual sampling – twice a week or weekly – 500 mL in plastic bottles | Monte carlo simulation –  SEIR model: susceptible, exposed, infectious, and recovered. | Assuming a 5 day of incubation (Wölfel et al., 2020) until the peak shedding rate on day 5 of infection. Contribution to viral load up to day 25 post infection.   - Shedding profile - Viral load   [Github](https://github.com/scwatson812/COVID19WastewaterModel)  [ShinyApp](https://rennertl.shinyapps.io/Wastewater_projections/) |
| 18 & 19 | (Melvin, Hendrickson, et al., 2021)  (Melvin, Chaudhry, et al., 2021) | 17 April 2020 to 31 Aug 2020 (4.5 months) | Single state in USA: population of ~5.4 M Minnesota + 6 surrounding regions | USA - Minnesota | 19 sites covering : state’s major metropolitan area | 570 samples from 19 participants. Composite samples 50-500 mL: once a week (start) and then once every 2-weeks (after first month) | Pearson correlation coefficient | Viral load  Values of Melvin’s Index (see the paper or github link)  Confirmed new covid-19 cases  [Github](https://github.com/glennesimmonsjr/dirtywatercooler)  Method for calculation of Melvin’s index – [R custom codes available](https://github.com/glennesimmonsjr/dirtywatercooler) |
| 20 | (Monteiro et al., 2022) | 27 April 2020 to 2 December 2020 (4 months) | Single city (or smaller): 204 COVID-19 hospitals | Portugal | 5 WWTPs : 404 samples | 24 hour composite sampling | No formal modelling | --- |
| 21 | (Murakami et al., 2020) | A letter to editor on 20^th^ April 2020 | | | | | | |
| 22 | (Olesen et al., 2021) | Wastewater based epidemiology for early warning signal | | | | | | |
| 23 | (Omori et al., 2021) | 22 March 2020 to 11 August 2020 | Single state:  Massachusetts | USA - Massachusetts | 1 WWTP: 3-7 times a week sampling | --- | Maximizing the likelihood function considering a Poisson sampling process.  Multiple regression analysis: to pin point heterogeneity between viral load and reported number of cases. | Concentration of a human fecal indicator  Age group incidence  Viral load |
| 24 | (Pájaro et al., 2022) | May 2020 to May 2021 | Single City: Galicia with predictions being at local level for small and medium size municipalities | Galicia - Spain | 11 WWTP  Population between 2000 and 23,000 | Autosamplers: 24 hours composite sample.  1-2 samples per week | SEIR model: susceptible, exposed, infectious, and recovered  model parameters:  constant recovery rate =1/14 (cumulated incident for a 14 days time interval)  Infection rate = estimated from public health data on infected individuals | Measurement of viral load in wastewater  Cumulative incident rate per 14 days |
| 25 | (Paterson & Durrheim, 2022) | No formal prediction modelling | | | | | | |
| 26 | (Pérez-Cataluña et al., 2022) | April 2020 and January 2021 | National: Spain | Spain | 14 WWTP: across the country | 76 grab samples | No formal models conducted |  |
| 27 | (Petala et al., 2021) | October 5th, 2020 until January 6th | Single city:  Thessaloniki – Greece (pop=700,000) | Greece | 1 WWTP serving the whole city of Thessaloniki | 24 hour composite samples (100ml hourly samples). | Model developed based on Wolf el al.’s work for estimation of shedding rate | Viral load |
| 28 | (Petros et al., 2022) | Autumn 2020 – Spring 2021 | Single University site | Colorado Mesa University - USA | 6 on-campus swage sites | Autosampler : 24 hours composite sample | Multiple linear regression | Demographics  Contact tracing  Wifi-based location data  Pathogen surveillance from wastewater  Diagnostic testing |
| 29 | (Pillay et al., 2021) | South Africa |  |  |  |  | Crude estimation of cases |  |
| 30 | (Proverbio et al., 2022) | Open data Up to August 2021 | Multi-country in Europe | 12 regional areas: Europe & North America | Not specific | COVID-19 Wastewater Analyser (CowastewaterAn)  Modified SEIR model with the Extended Kalman Filter. | SEIR stochastic model  A dynamic epidemiological model | Data & code: [https://gitlab.lcsb.uni.lu/SCG/cowastewateran](https://gitlab.lcsb.uni.lu/SCG/cowwan) |
| 31 | (Srinivas et al., 2021) | Not applicable | 13 states in USA | USA | Not applicable | Auto-sampler will be installed at targeted locations identified by the fuzzy model | Serial connection network.  Fuzzy-Bayesian optimization model | Urban, Rural demographics  Migration rate (high/low)  Quarantine facilities (good, pool)  Strict regulations(Yes, No)  Education(poor, good)  Healthcare facilities (good, pool)  Temperature and weather conditions (below 10, 10-20, 20-30 and above30)  Population density (low, high)  Population demography (male, female)  Age-bands (<18, 19-29, 30-49, 50,84, 85+)  Comorbidities (Respiratory, kidney, obesity, hypertension and none) |
| 32 | (Saguti et al., 2021) | Feb 10^th^ – July 5^th^-2020 | Single city: Gothenburg | Sweden | 1 WWTP (Rya) | Fixed site autosampler | None-model based |  |
| 33 | (Saththasivam et al., 2021) | 21^st^ and 30^th^ August 2020 | Single city: Doha | Qatar | 5 WWTPs across the Doha | N=43 samples  Raw composite samples (2h frequency intervals over 24hour) |  |  |
| 34 | (Spurbeck et al., 2021) | Feasibility study of tracking COVID-19 through wastewater | | | | | | |
| 35 | (Vallejo et al., 2021) | 15 April – June 4^th^ – 2020 | Regional: Galicia region | Spain | 1 WWTP serving the Coruna metropolitan area | Composite samples – autosampler with collecting samples over 24 hour every 2 hours. | Generalized Additive Models and the Locally Estimated Scatterplot Smoothing (LOESS) | Viral load |
| 36 | (Vaughan et al., 2023) | Variable per region – information available at (<https://sphere.waterpathogens.org/map> ) | Multi regions | 108 Cities in Five Countries (Scotland, Catalonia, Ohio, the Netherlands, and Switzerland) | For each region information are available at (<https://sphere.waterpathogens.org/map> ) | For each region information are available at (<https://sphere.waterpathogens.org/map> ) | Machine Learning (Random Forest – 100 trees, 80% training set and one step ahead prediction based on sampling frequency on each WWTP that is automatically detected from each dataset) | Sampling frequency  Flow rate  Viral incubation period  Viral loads |
| 37 | (Vo et al., 2022) | March 2020 – April 2021 | Regional: Southern Nevada | USA | 8 WWTP |  |  |  |
| 38 | (Zhao et al., 2022) | 1 September 2020 | 4 October 2021 | Multi regions | City of Detroit, and Wayne, Macomb, Oakland | 1 wastewater authority in southeast Michigan (407 samples) | VIRADEL sampling method  &  24-hour composite sample | Autoregression models |
| 39 | (G. Li et al., 2023) | 1 June 2021 | 30 March 2022 | Lower super output areas (LSOAs) in England | England- UK | 303 Sewer Treatment Plants  32,844 LSOA | Autosampler: 24 hours composite sample info provided in separate paper by (Hillary et al., 2021) | Spatially continuous model using Bayesian modelling framework |

Table S3- Categorisation of the modelling approaches in five themes – each theme is color coded accordingly

| **No.** | **Theme** | **Authors** | **Models** |
| --- | --- | --- | --- |
|  |  |  |  |
| 1 | Linear regression and time series analysis | Krivoňáková et al 2021 (Krivoňáková et al., 2021) | Regression models: relationship of wastewater data and COVID-19 case count  Time-series analysis: wastewater time series and various time lags of positive RT_qPCR test and COVID deaths.  Applied a cross correlation function to identify the best match.  GAM model used for illustration of the smoothed curves of time-series. |
| 2 | Bayesian models | Srinivas et al 2021 (Srinivas et al., 2021) | Serial connection network.  Fuzzy-Bayesian optimization model |
| 3 | Linear regression and time series analysis | Omori et al. (Omori et al., 2021) | Maximizing the likelihood function considering a Poisson sampling process.  Multiple regression analysis: to pinpoint heterogeneity between viral load and reported number of cases. |
| 4 | Compartmental SEIR models and agent-based models | McMahan et al. 2021 (McMahan et al., 2021) | Monte Carlo simulation –  SEIR model: susceptible, exposed, infectious, and recovered  (model parameters: Median incubation period (fixed)=0.2=5 days  . |
| 5 | Compartmental SEIR models and agent-based models | Amereh et al. 2021 (Amereh et al., 2021) | Monte Carlo Simulation |
| 6 | Linear regression and time series analysis | Galani et al. (Galani et al., 2022) | Linear regression model of time-series data.  Multi-layer artificial neural network (ANN) using backpropagation algorithm.  Bayesian Distributed-Lag non-linear model of Poisson family with log-link (positive regression coefficient from gamma distribution with unknown shape and scale) |
| 7 | Compartmental SEIR models and agent-based models | Scottish Government (gov.scot, 2022b) | Two approaches for predicting R number:  1-Using wastewater derived data  2-Using data from an agent-based model |
| 8 | Linear regression and time series analysis | Fitzgerald et al. (Fitzgerald et al., 2021) | 1-Linear regression for prediction of daily concentration from independent variables:catchment population and site  2-Spearmen’s rank correlation between viral concentration and number of positive cases  3-Linear mixed model: with fixed coefficients for daily viral load and random intercept and coefficients for each catchment. |
| 9 | Compartmental SEIR models and agent-based models | Proverbio et al. 2022 (Proverbio et al., 2022) | COVID-19 Wastewater Analyser (CowastewaterAn)  Modified SEIR model with the Extended Kalman Filter. |
| 10 | Spatio-temporal models | Kuhn et al. 2022 (Kuhn et al., 2022) | Spatio-temporal model of total daily cases across sewersheds was automated using Esri’s ModelBuilder. Spatial census areas from Esri’s ArcMap and QGIS were used to quality control the overlayed polygon for sewershed areas.  GLM models: viral load & incident COVID-19 cases (per 100,000). |
| 11 | Compartmental SEIR models and agent-based models | Pájaro et al 2022 (Pájaro et al., 2022) | SEIR model: susceptible, exposed, infectious, and recovered |
| 12 | Linear regression and time series analysis | Petros et al. 2022 (Petros et al., 2022) | Multiple linear regression |
| 13 | Spatio-temporal models | (G. Li et al., 2023) | Spatially continuous model using Bayesian modelling framework |
| 14 | Autoregression model | (Zhao et al., 2022) | Autoregression models based on RNA viral load concentration |
| 15 | Machine learning models | Vaughan et al. 2022 (Vaughan et al., 2023) | Machine Learning (Random Forest – 100 trees, 80% training set and one step ahead prediction based on sampling frequency on each WWTP that is automatically detected from each dataset) |
